# Diet Quality during Infancy and Early Childhood in Children with and without Risk of Type 1 Diabetes: A DEDIPAC Study

**DOI:** 10.3390/nu9010048

**Published:** 2017-01-09

**Authors:** Stefanie Schoen, Sibille Jergens, Janett Barbaresko, Ute Nöthlings, Mathilde Kersting, Thomas Remer, Marta Stelmach-Mardas, Anette-G. Ziegler, Sandra Hummel

**Affiliations:** 1 Research Institute of Child Nutrition, University Clinic Bochum, Bochum 44791, Germany; S-Schoen@gmx.net (S.S.); kersting@fke-do.de (M.K.); 2 Institute of Diabetes Research, Helmholtz Zentrum München, and Forschergruppe Diabetes, Klinikum rechts der Isar, Technische Universität München, Neuherberg 85764, Germany; sibille.jergens@hotmail.com (S.J.); anette-g.ziegler@helmholtz-muenchen.de (A.-G.Z.); 3 Forschergruppe Diabetes e.V., Neuherberg 85764, Germany; 4 German Center for Diabetes Research (DZD), München-Neuherberg 85764, Germany; 5 Department of Nutrition and Food Sciences, Nutritional Epidemiology, University of Bonn, Bonn 53115, Germany; j.barbaresko@uni-bonn.de (J.B.); noethlings@uni-bonn.de (U.N.); 6 DONALD Study Dortmund, Department of Nutrition and Food Sciences, Nutritional Epidemiology, University of Bonn, University Branch Dortmund, Dortmund 44225, Germany; remer@uni-bonn.de; 7 Department of Epidemiology, German Institute of Human Nutrition Potsdam-Rehbruecke, Nuthetal 14558, Germany; marta.stelmach@dife.de; 8 Department of Pediatric Gastroenterology and Metabolic Diseases, Poznan University of Medical Sciences, Poznan 60-572, Poland

**Keywords:** nutrient intake quality, food intake quality, infancy, early childhood, type 1 diabetes

## Abstract

Previous studies have indicated that mothers of children at increased risk of type 1 diabetes (T1D) may modify their child’s diet following risk notification. Our aim was to investigate the diet quality after notified of T1D risk in at-risk children compared to not-at-risk children. The quality of nutrient intake (PANDiet score) and food intake (analyzed by a newly developed score and the HuSKY score) were assessed using three-day dietary records collected for at-risk children (BABYDIET study, *n* = 109) and a matched sample of not-at-risk children (DONALD study, *n* = 205) at nine and 24 months of age. Nutrient and food intake quality were lower at nine months of age and food intake quality was lower at 24 months of age in at-risk than in not-at-risk children (*p* = 0.01 and *p* < 0.0001, respectively). The amount of added sugar was higher in at-risk children at both ages (*p* < 0.0001). In at-risk children, dietary quality was similar between children who were first exposed to gluten at six or 12 months of age. Despite being notified about their child’s risk of T1D, the child’s mother did not switch to healthier diets compared with not-at-risk mothers.

## 1. Introduction

Type 1 diabetes (T1D), a chronic inflammatory disease caused by a selective destruction of the pancreatic β-cells, is one of the most common and serious chronic diseases in children with around 86,000 newly diagnosed children each year [1,2]. Although the pathogenesis of islet autoimmunity and type 1 diabetes is not yet fully understood, a genetic susceptibility is well documented, and environmental factors, such as early dietary factors, are thought to act as potential initiating exposures in the pathogenesis [1]. Most of the research on early dietary factors and islet autoimmunity or T1D risk has focused on the role of breastfeeding duration and the age at introducing complementary feeding [1]. While the majority of the prospective cohort studies could not show an association of breastfeeding duration with islet autoimmunity and/or T1D risk, two prospective cohort studies reported an association of timing of first exposure to gluten [3] or any type of cereals [4] with islet autoimmunity. These results led to the initiation of BABYDIET study [5], a dietary intervention study in which newborns at increased risk of developing T1D were randomized to either gluten introduction at 12 months (intervention group) or at six months (control group) of age. In this study, delayed gluten introduction did not reduce the risk of developing islet autoimmunity [5]. However, participation in a dietary intervention study may have an impact on overall nutrient and food intake quality, although the intervention is limited to gluten-containing food. In a previous analysis of the BABYDIET cohort, we did not reveal differences of breastfeeding duration and timing of solid food introduction between children in the BABYDIET intervention and control group [5]. However, adherence to a gluten-free diet was recently reported to be associated with inadequate nutrient intake in a survey of patients with celiac disease [6].

Due to the observed inconsistencies between studies addressing early dietary factors and islet autoimmunity risk [1,7], no specific dietary recommendations are available for infants at increased risk of T1D. Still, many families indicate to implement behavioral changes after being notified that their child is at increased risk of T1D in order to prevent the child developing the disease [8,9,10,11]. Changes in dietary behavior, such as reducing the child’s sugar or carbohydrate intake, were the most common types of behavioral changes to be reported [11]. However, most studies were based on questionnaires containing a list of possible actions to prevent T1D and only few studies were performed using dietary data to assess differences between infants whose families were notified about the increased risk of T1D and infants of the general population [12,13]. These studies observed shorter breastfeeding duration in newborns at increased risk of T1D when compared to not-at risk newborns [12], while no differences in dietary patterns were observed between at risk and non-at risk children at 8–12 years of age [13].

Considering the putative role of the child’s diet on the development of islet autoimmunity, we therefore sought to investigate whether dietary intervention in the first year of life affected overall dietary quality up to the age of 24 months, and whether this might bias BABYDIET analyses using islet autoimmunity and T1D as endpoints. To achieve this, we aimed to determine whether nutrient and food intake quality differed at nine months (during dietary intervention) and 24 months (one year after dietary intervention) between children of the BABYDIET study, who were randomized to the dietary intervention group and children randomized to the control group. Secondly, we aimed to compare nutrient and food intake quality between children, whose families were notified of an increased risk of T1D (at-risk children, BABYDIET) and children who are not at increased risk of T1D (not-at-risk children) participating at the German observational DONALD study.

## 2. Methods

### 2.1. Study Design and Subjects

Data from two ongoing German prospective studies were used in this analysis. The BABYDIET study was performed to investigate whether delaying exposure to gluten could reduce the risk of developing islet autoantibodies in children who are genetically at risk for T1D [5]. The DONALD study is an ongoing open cohort study that is collecting data on diet, growth, development, and metabolism from early infancy until adulthood [14]. The studies were homogeneous in terms of their early follow-up designs and application of dietary assessment methods.

The BABYDIET study enrolled 150 newborns with at least one first-degree relative with T1D between 2000 and 2006, as previously described [5]. Infants of the BABYDIET study were recruited German-wide and were eligible if they were younger than 2 months of age, not yet exposed to gluten and had at least two first-degree relatives with T1D or one first-degree relative with T1D and one of the HLA genotypes *DR3/4-DQ8*, *DR4-DQ8/DR4-DQ8*, or *DR3/3*. Infants were excluded from the study if they had an illness or birth defect that precludes long-term follow-up. Written informed consent for genetic screening as well as for enrollment into the intervention trial after notification of increased T1D risk was provided by the infant’s parents. After inclusion, newborns were randomized to one of two groups in which gluten was to be introduced at 12 months (intervention group) or at 6 months (control group) of age. Furthermore, each participating family was visited by a dietitian who explained the gluten-free diet. Parents were provided lists with the most common foods introduced during the first year of life, and gluten-containing products were indicated. Details of gluten-free commercial infant products were provided to the parents as well. Children were followed up every 3 months until 3 years of age and yearly thereafter to detect islet autoimmunity and to assess dietary intake, growth, and metabolism. The BABYDIET study is being conducted at the Institute of Diabetes Research, Helmholtz Zentrum München, Munich, Germany, and was approved by the ethics committee at Ludwig-Maximilians University, Munich, Germany (No. 329/00).

Starting in 1985, the DONALD study recruited >1500 apparently healthy children in the city of Dortmund and surrounding areas, as previously described [14]. Eligible were healthy (no prevalent diseases affecting growth and/or diet) German babies (age 3–6 month) whose mothers and/or fathers were willing to participate in a long-term study and of whom at least one parent had sufficient knowledge of the German language. Follow-up visits were scheduled every 3 months for the first year, bi-annually in the second year, and annually thereafter to assess dietary intake. The DONALD study is being conducted by the University of Bonn, Germany, and was approved by the ethics committee of the University of Bonn, Germany (No. 098/06). Written informed consent was obtained from the parents.

**Figure A1:**
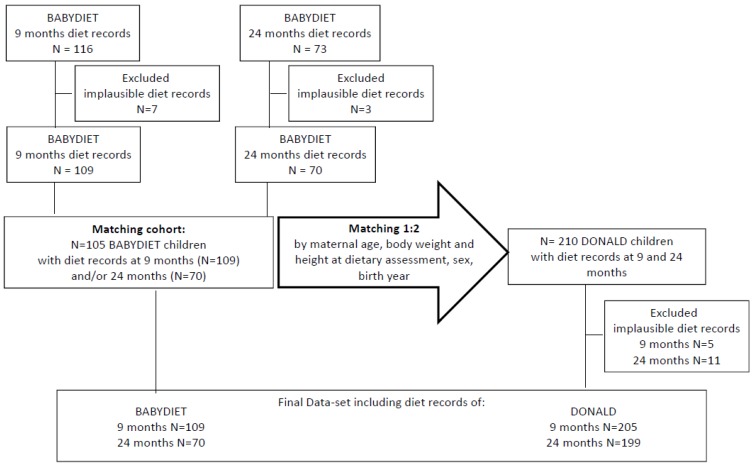
Flow-diagram of study cohort.

In the BABYDIET study, dietary intake data were available for 116 children at 9 months of age (during dietary intervention) and in 73 children at 24 months of age (minimum of 6 months after the dietary intervention, Figure A1). Seven children were excluded from the analysis at 9 months and 3 at 24 months of age because of implausible dietary records. Therefore, the at-risk population comprised 109 children at 9 months of age and 70 children at 24 months of age. All of the at-risk children with plausible data on dietary intake and complete data on body weight and height at the dietary assessment, sex, birth year, and maternal age (*n* = 105) were matched with two not-at-risk children in the DONALD study based on these variables. Therefore, dietary intake data were available for 210 not-at-risk children at 9 months and 24 months of age. However, 5 children were excluded from the analysis at 9 months and 11 at 24 months of age because of implausible dietary records.

### 2.2. Dietary Records

In both studies, dietary intake was assessed by 3-day weighed dietary records, as previously described [5,15]. In the BABYDIET study, three-day weighed dietary records were collected 3-monthly from 3 months until 3 years of age and in the DONALD study 3-monthly from 3 months until 12 months of age, bi-annually until 2 years of age and annually thereafter. In both studies, parents were instructed to weigh and record all foods and beverages consumed by their child using electronic food scales on three consecutive days, preferably including two week-days and one week-end day. Recipes (ingredients and preparation) of homemade food as well as a detailed description including type and brand of commercial foods were requested. If exact weighing was not possible, semi-quantitative recording with household measures (e.g., number of glasses, cups, and spoons) was allowed. For children of the BABYDIET study, who received breast milk during the dietary recording, the mothers were asked to record each breastfeeding meal and amounts of breastmilk were estimated from calculated energy requirement [16]. For children of the DONALD study, parents were provided a baby scale and advised to record baby’s weight before and after breastfeeding and the breast milk amount was calculated by the weight difference. The 3-day weighed dietary records from BABYDIET children were sent by mail to the clinical center and checked for accuracy and completeness by a trained dietitian located at the DONALD study site. In the DONALD study, a trained dietitian visited the family at home and checked the records for accuracy and completeness.

All dietary records were entered and analyzed using the continuously updated in-house nutrient database, LEBTAB, which incorporates information from standard nutrient tables or recipe simulation based on the labelled ingredients and nutrients, including fortification of nutrients [15]. The individual daily intakes of energy, nutrients, and food groups were calculated as arithmetic means of the three daily records. The present analysis only included plausible dietary records after excluding underreporting, which was calculated as the ratio of energy intake (kcal) to the basal metabolic rate (BMR). Dietary records with a EI/BMR ratio of <0.97 were excluded [17].

### 2.3. PANDiet Score

**Figure A2:**
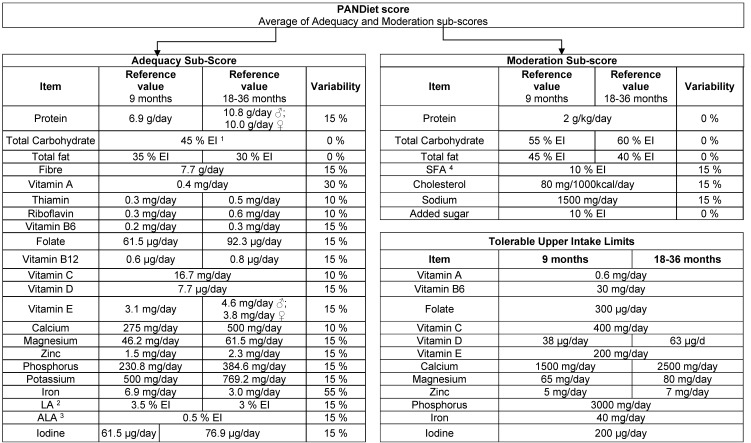
Determination of the PANDiet score in the BABYDIET and DONALD studies. ^1^ EI = Energy intake; ^2^ LA = Linoleic Acid; ^3^ ALA = Alpha Linolenic Acid; ^4^ SFA = Saturated Fatty Acids.

The PANDiet score was developed to assess the overall dietary quality of an individual in terms of the probability of having an adequate nutrient intake [18], and was recently adapted for and evaluated in young children in the United Kingdom [19]. The PANDiet score is based on two scores, Adequacy and Moderation, and takes into account the duration (in days) of dietary assessment, the mean intake and the day-to-day variability in intake, the nutrient reference value and the inter-individual variability of intake. The PANDiet score is calculated as the mean of the Adequacy and Moderation sub-scores, and ranges from 0 to 100, where higher score indicate better diet quality. For the present analysis, the PANDiet score calculation was adjusted according to the current national reference values for nutrient intake in children [20] and the nutrients available in LEBTAB (Figure A2).

### 2.4. Added Sugars

Added sugars were defined as sugars and syrups that are added to foods during processing or preparation. Specifically, added sugars include white sugar, brown sugar, raw sugar, corn syrup, corn syrup solids, high-fructose corn syrup, malt syrup, maple syrup, pancake syrup, fructose sweetener, liquid fructose, honey, molasses, anhydrous dextrose, and crystal dextrose. Half of the weight of maltodextrin was included because it consists of a mixture of different mono-, di-, and oligosaccharides. Lactose used as an ingredient in infant formula, follow-on formula or growing-up milk was not considered as added sugars because this is the natural occurring carbohydrate in the prototype breast milk.

### 2.5. Quality of Food Intake at 9 Months

The quality of dietary intake in terms of food groups and meal level was assessed using two different approaches in order to address the differences in dietary recommendations and eating patterns between children aged 9 and 24 months. To our knowledge, there are no score to assess the quality of food intake at 9 months of age relative to national recommendations. Therefore, we developed a new score in this study, termed the Score Of Compliance to Complementary fEeding Recommendations (SOCCER). To calculate the score, the types of meals were classified as either commercial dishes or by individual meal components recorded as one meal from the 3-day dietary records. Meals were categorized according to German food-based dietary guidelines [21], as shown in Table A1. Incomplete meals, such as vegetables only or beverages, and dishes that were not mentioned in the recommendations, such as snacks, were not included in the score calculation. The mean number of meals per day was calculated for each category. To calculate cow’s milk intake, the mean daily intake (g) of milk and dairy products was calculated, with the assumption that 15 g of hard cheese and 30 g of soft cheese corresponds to 100 mL of milk. Commercial products for infants, which need to be reconstituted with water (dry food), such as infant formula and infant cereals with milk powder, were ignored from the calculation. Industrial products for infants (wet food), including yoghurt, were assumed to have a milk content of 30%. If the meal type was not given during the 3-day period, the mean number was set to 0.

For each meal type, the observed mean intake was related to the recommendations by calculating the ratio (I/R) = intake (I) of meal type X/recommendation (R) for meal type X. For this comparison, the “intake of meal type” was expressed as the number of portions from the specific meal types per day or, in the case of cow’s milk, as mL/day. Next, the ratio (I/R)X was allocated a number of points, relative to the recommended intake (in percent) of that meal type ((I/R)X → scoreX).

For all meal types, except cows’ milk, intake less than the recommended number of meals was assessed proportionally. For example, it is recommended to daily feed a meal composed of vegetables, potatoes and meat. If this meal type was only fed two times within 3 days, 66 points were allocated for this specific meal type. However, we did not account for intake exceeding the recommendation. The points for each meal type were summed together and standardized on a scale ranging from 0 to 100 (SOCCER = ∑scoreX/number of scoreX), where higher scores indicate better overall feeding patterns.

### 2.6. Quality of Food Intake at 24 Months

The Healthy nutrition Score for Kids and Youth (HuSKY), which was developed by Kleiser et al. for the German KiGGS study [22], was used to assess the quality of food intake at 24 months. In brief, the mean daily intake of food groups, which are defined in the concept of the Optimized Mixed Diet (OMD), was related to the age-specific daily intake recommendations of the OMD [21,23]. The ratio of the consumed to recommended intake was allocated points for all food groups, which were summarized and standardized on a scale ranging from 0 to 100 as described in detail by Kleiser et al. [22].

### 2.7. Matching Variables

Body weight and height were measured at each dietary assessment visit. In the BABYDIET study, weight and height were measured by a physician during the clinical study visits. In the DONALD study, weight and height were measured by trained and regularly monitored nurses who followed standard procedures. Using these data, the body mass index (BMI) was calculated. The child’s year of birth, sex, and the maternal age were collected by questionnaires in both studies.

### 2.8. Covariates

In both studies, the maternal educational level was determined by a questionnaire when the children were 3 months old. In both studies, maternal education was recorded as highest school education achieved, categorized into no certificate, secondary general, secondary intermediate school certificate, and higher education entrance certificate. For this analysis, maternal educational level was re-coded as low (secondary general or intermediate school certificate) or high (higher education entrance certificate). Information on maternal educational level was missing in 35 children at 9 months and 23 at 24 months of age in the BABYDIET cohort and in 1 child in the DONALD cohort. Because of the extent of missing data in the BABYDIET cohort, maternal educational level was not used as a matching variable.

### 2.9. Statistical Analysis

The median and interquartile range (IQR) were calculated for the PANDiet, HuSKY, and SOCCER scores, and the intake of added sugar. The scores and intake of added sugar were compared between the BABYDIET intervention and control groups, between children of mothers with T1D and children of fathers/siblings with T1D and between the BABYDIET and DONALD study cohorts using the Mann–Whitney *U* test to detect statistically significant differences. For the comparison of nutrient and food intake quality between the BABYDIET and DONALD study cohorts, data were further stratified by maternal educational level. Associations between the PANDiet score and food intake scores were explored using Pearson’s correlation coefficients. Nutrient and food intake quality was also assessed in relation to maternal age. To achieve this, the median age of mothers at the time of collecting dietary information was calculated and the maternal age was coded as either higher than or equal to/lower than the median. The median maternal age was 33.4 years and 34.8 years for dietary information collected at 9 and 24 months of age, respectively.

For all analyses, the significance level was set to 0.05. All calculations were carried out using IBM SPSS Statistics 24 (IBM Corp., Armonk, NY, USA).

## 3. Results

### 3.1. Baseline Characteristics of the BABYDIET and DONALD Study Cohorts

Characteristics of children of the BABYDIET and DONALD study cohorts included in this analysis are shown in Table 1. Owing to the matching design, the children’s age and BMI, and the maternal age at dietary assessment were similar in both study cohorts. However, a greater proportion of mothers in the DONALD cohort had obtained a higher education entrance certificate than mothers in the BABYDIET cohort.

### 3.2. Associations of Dietary Intervention and Having a Family Member with T1D on Nutrient and Food Intake Quality in the BABYDIET Cohort

In the BABYDIET cohort, there were no significant differences in nutrient or food intake quality or the amount of added sugar at nine or 24 months of age between the control and intervention groups.

The PANDiet score at nine months of age was lower in children with a father and/or sibling with T1D than in children with a mother with T1D (*p* = 0.004, Table 2).

None of the children had developed T1D before or at dietary assessment.

### 3.3. Comparison of Nutrient Quality and Food Intake Quality between Children of the BABYDIET and DONALD Cohorts

At nine months of age, nutrient quality (PANDiet score) and food intake quality (SOCCER score) were significantly lower in the BABYDIET cohort than in the DONALD cohort (PANDiet, *p* = 0.01; SOCCER: *p* < 0.0001; Table 3). At 24 months of age, food intake quality (HuSKY score) was significantly lower in the BABYDIET cohort than in the DONALD cohort (*p* < 0.0001; Table 3). Differences in the HUSKY score between the cohorts could not be attributed to differences in the intake of individual food groups, and instead was probably due to less favorable intake of multiple food groups (Table 4). The intake of added sugar was significantly greater in the BABYDIET cohort than in the DONALD cohort at nine months and at 24 months (*p* < 0.0001, Table 3). In the total cohort of children (BABYDIET plus DONALD), nutrient quality was significantly correlated with food intake quality at nine and 24 months of age (*r* = 0.3, *p* < 0.01 for both age groups).

### 3.4. Effects of Maternal Educational Level and Maternal Age on Nutrient and Food Intake Quality

Because a greater proportion of mothers in the DONALD study had obtained a higher education entrance certificate than mothers in the BABYDIET study, the nutrient and food intake data were analyzed with stratification according to maternal educational level (Table 5). In both strata, the differences in nutrient and food intake quality between the BABYDIET and DONALD cohorts were consistent with those in the total cohort, although the differences were more pronounced in the higher maternal education stratum.

We also examined the influence of maternal age on nutrient and food intake quality by dividing the children into groups according to the maternal age at the time of data collection. In this analysis, the intake of added sugar was greater in children of younger mothers than in children of older mothers (≤33.4 vs. >33.4 years, median (IQR): 5.0 (1.1–13.2) vs. 2.2 (0–6.9) g/day; *p* < 0.001). 

## 4. Discussion

Nutrient and food intake quality was not affected by dietary intervention in children participating at the BABYDIET study. However, among BABYDIET children nutrient quality was greater in the children of mothers with T1D than in the children of non-diabetic mothers at nine months of age. Compared to not-at risk children in the DONALD study, dietary quality was significantly lower in children at increased risk of developing T1D in the BABYDIET study. In particular, the BABYDIET cohort was characterized by lower nutrient and food intake quality at nine months of age, lower food quality at 24 months, and increased intake of added sugar at nine and 24 months of age.

Adherence to a gluten-free diet is challenging and a previous published survey in adult patients with celiac disease reported higher fat and lower carbohydrate intake in females and lower intake of several vitamins and minerals in male and female adult patients [6]. Our results do not indicate that nutrient and food intake quality was affected by the dietary intervention, neither during the intervention period where families were advised to avoid gluten in their child’s diet, nor 12 months after the intervention period.

In contrast, nutrient quality at nine months of age was greater in the children of mothers with T1D than in children with a father/sibling with T1D, although food intake quality did not significantly differ between these two groups. In a previous analysis of the BABYDIET study, the duration of breastfeeding was lower in the children of mothers with T1D [24]. This may be due to a higher frequency of neonatal complications in pregnancies complicated by T1D [25]. It may be that mothers put more effort into healthy, complementary feeding habits to compensate for the shorter breastfeeding duration. Our results further indicate that dietary quality is lower in children, whose families were notified of an increased risk of T1D than in not-at risk children. Previous studies have been reporting behavioral changes towards healthier diets among families who were notified that their child is at increased risk of developing T1D [8,9,10,11]. In these studies, about 30% of families reported that they changed the diet of their child to prevent it from developing T1D, for example by reducing the consumption of sweets and carbohydrates [11]. By contrast, our study revealed that the at-risk children in notified families consumed higher amounts of added sugar at nine and 24 months and had lower nutrient and food intake quality than not-at-risk children. Previous reports examining the changes in dietary behaviors involved interviews in which the mothers were asked to indicate on a list if they did anything to try to prevent T1D in their child, and did not include detailed dietary intake data [9,10,11]. Based on our findings we hypothesize that mother’s intention to change dietary behavior is not necessarily reflected in her actual behavior.

Socioeconomic status was reported to affect dietary quality in children in previous studies [22] and the DONALD cohort is characterized by a relatively high educational level [14]. Indeed, a greater proportion of mothers in the DONALD cohort had obtained the highest educational level compared with mothers in the BABYDIET cohort. To exclude the possibility that the differences in dietary quality between the BABYDIET and DONALD cohorts were due to the higher maternal education level in the DONALD cohort, we stratified both cohorts according to the mother’s educational level. Most of the differences in dietary quality between the BABYDIET and DONALD cohorts before stratification were also observed after stratification by education level, indicating that the differences in dietary quality are independent of maternal educational level. However, we cannot exclude that other, unmeasured, variables, including other socioeconomic variables, are associated with diet quality and act as confounders in this analysis.

Several nutritional epidemiological studies have investigated nutrient or food intake quality during infancy and early childhood in the general population by applying indices based on nutrient or food intake [19,26,27]. One recent study in the United Kingdom evaluated the PANDiet score in children aged 12–18 months, and included 25 nutrients, of which four were different from those included in our study [19]. The authors applied a four-stage strategy to evaluate the content and construct validity of the PANDiet score in this population and concluded that the PANDiet is a valid tool to assess diet quality in young children. The mean PANDiet score in that study was 70.2, which is greater than the scores in both of our cohorts. However, direct comparison of the score is limited owing to the different national reference intake values, which were used to calculate the score, and the inclusion of different nutrients.

Thus far, we are unaware of any studies using a score to assess meal-based food intake quality during the complementary feeding period. The German recommendations for complementary feeding are very detailed with respect to the composition of recommended meals and the tolerated intake of cow’s milk or dairy products as part of an infant’s diet [28]. We used these recommendations to develop the food quality score (SOCCER). By applying this score, we found high compliance to the recommendations in the DONALD cohort, and food intake quality at nine months of age was greater in the DONALD cohort than in the BABYDIET cohort. This may be explained by more individual counseling of the DONALD participants at study entry. However, a limitation of the SOCCER score is that it is solely based on the types of meals and the components of meals, but ignores meal portion sizes. Additionally, meals or snacks, which are not mentioned in the dietary guidelines, as well as ingredients that have been added to the meal beside the score ingredients (e.g., added sugar or fat), were not considered in the calculation. Therefore, the SOCCER score may overestimate food intake quality.

Food intake quality at 24 months was comparable between children in the DONALD study and 3–6-year-old children in the German KiGGS study [22] with mean HuSKY scores of 58.6 and 59.3, respectively. Both of these studies were performed in the general population and used the same score (HuSKY) to determine food intake quality. However, this comparison should be made cautiously because different dietary assessment methods were used. The DONALD and BABYDIET studies used three-day dietary records, which may underestimate the intake of foods that are consumed less frequently (e.g., fish and eggs) compared with food frequency questionnaires, which were used in the KiGGS study. The lower HuSKY score in the BABYDIET cohort may reflect less favorable intake of all food groups rather than individual food groups. Similar findings were reported by Verger et al. who analyzed PANDiet scores in relation to the intake of different food groups [19].

The main strength of this study is that we analyzed dietary quality in a prospective cohort of children at increased risk of developing T1D and compare dietary quality to that observed in a prospective cohort of not-at-risk children, in which the dietary data were collected, processed, and analyzed using the same protocol. The longitudinal approach of both studies enabled us to investigate dietary quality at two time-points in the child’s early life, a time considered to be important in long-term health development either through programming mechanisms or through developing food-specific preferences. Additionally, we used weighed dietary records which are regarded to have the least correlated errors among common dietary assessment methods and may thus also be used as a standard to assess validity of food frequency questionnaires [29,30]. A limitation of our study is that our results are based on data for only 109 children in the BABYDIET study. Most of the participating children had German parents; therefore, our findings may not be generalizable to other countries and/or ethnicities.

## 5. Conclusions

In the BABYDIET cohort, dietary quality was not significantly different between children who were assigned to the dietary intervention group (first gluten exposure delayed until 12 months of age) and the control group. Furthermore, our study does not indicate that families who are notified that their child is at increased risk of developing T1D provide their child with a healthier diet than families without family history of T1D. Therefore, it is unlikely that study characteristics, such as dietary intervention and risk notification, are associated with overall dietary quality during the first 24 months, and are unlikely to introduce bias into the outcome analysis of the BABYDIET study.

## Figures and Tables

**Table 1:** Characteristics of the study cohorts.

	BABYDIET Cohort		DONALD Cohort	
	9 Months	24 Months	9 Months	24 Months
3-day dietary records	109	70	205	199
Age of child, months *	9.1 (8.9–9.5)	24.4 (22.3–35.9)	9.2 (9.0–9.5)	23.9 (20.7–24.8)
Birth year, IQR	2003–2005	2002–2010	2002–2005	2002–2009
Females	65 (59.6)	43 (61.4)	123 (60.0)	118 (59.3)
Dietary intervention group	54 (49.5)	37 (52.9)	-	-
Mother with T1D	55 (50.5)	38 (54.3)	-	-
Father or sibling with T1D	54 (49.5)	32 (45.7)	-	-
BMI of child, kg/m^2^	16.5 (15.7–17.5)	15.8 (15.1–17.2)	16.9 (16.2–17.8)	15.9 (15.0–16.7)
Maternal age, years	32.6 (30.5–36.0)	34.6 (31.9–37.3)	33.6 (31.3–36.4)	34.9 (32.6–37.8)
Maternal educational level ^†,‡^				
Secondary general school certificate	3 (4.1)	-	1 (0.5)	1 (0.5)
Intermediate school certificate	31 (41.9)	19 (40.4)	25 (12.3)	25 (12.6)
Higher education entrance certificate	40 (54.1)	28 (59.6)	178 (87.3)	172 (86.9)

Data are presented as n (%) or median (IQR); * At dietary assessment; ^†^ Maternal educational level was determined when their children were three months old; ^‡^ Information on maternal education was not available in 35 children at nine months and 23 children at 24 months of age in the BABYDIET cohort. IQR, Interquartile Range; T1D: type 1 diabetes; BMI: body mass index.

**Table 2:** Nutrient and food intake quality in children aged nine and 24 months according to dietary intervention and family history of type 1 diabetes in the BABYDIET cohort.

	Dietary Intervention			Family Member with Type 1 Diabetes		
	Control	Intervention	*p*-Value	Father/Sibling	Mother	*p*-Value
9 months						
Nutrient quality						
PANDiet	65.1 (59.3–71.1)	66.3 (57.1–70.4)	0.9	63.7 (54.1–68.4)	67.7 (62.5–72.0)	0.004
Added sugar (g/day)	6.4 (2.3–16.9)	9.3 (2.1–21.2)	0.3	5.7 (1.6–13.7)	9.8 (3.4–20.9)	0.1
Food quality						
SOCCER	73.3 (60.0–80.0)	73.3 (60.0–81.7)	0.6	73.3 (60.0–80.0)	73.3 (60.0–86.7)	0.9
24 months						
Nutrient quality						
PANDiet	58.2 (54.4–62.6)	58.8 (55.7–61.3)	0.7	59.0 (53.4–62.2)	58.2 (55.4–61.5)	0.9
Added sugar (g/day)	27.0 (17.9–39.0)	32.4 (20.4–51.5)	0.1	26.8 (18.8–44.2)	31.9 (17.9–42.9)	0.9
Food quality						
HuSKY	54.6 (44.2–59–8)	46.9 (39.8–58–9)	0.2	52.7 (43.8–59.1)	51.9 (41.0–59.2)	0.7

**Table 3:** Nutrient and food intake quality of children at nine and 24 months of age in the BABYDIET and DONALD cohorts.

	BABYDIET Cohort	DONALD Cohort	*p*-Value *
9 months			
Nutrient quality			
PANDiet	65.9 (58.2–71.0)	68.3 (62.3–73.3)	0.01
Added sugar (g/day)	8.0 (2.2–19.1)	1.8 (0.1–6.7)	<0.0001
Food quality			
SOCCER	73.3 (60.0–80.0)	93.3 (80.0–100.0)	<0.0001
24 months			
Nutrient quality			
PANDiet	58.5 (54.9–61.7)	59.7 (55.6–63.5)	0.1
Added sugar (g/day)	30.0 (18.1–42.7)	18.7 (10.4–25.9)	<0.0001
Food quality			
HuSKY	52.5 (42.7–58.7)	58.6 (50.1–65.3)	<0.0001

Data are presented as the median (IQR). * Mann–Whitney *U* test. PANDiet: Probability of Adequate Nutrient Intake; SOCCER: Score Of Compliance to Complementary fEeding Recommendations; HuSKY: Healthy nutrition Score for Kids and Youth.

**Table 4:** Age-specific recommendations on daily food intake and the median intake of foods in children aged 24 months.

	Recommended Intake *	BABYDIET Cohort (*n* = 70)	DONALD Cohort (*n* = 199)
Beverages (mL/day)	700	429 (277–633)	466 (270–628)
Vegetables (g/day)	150	47 (31–86)	65 (38–97)
Fruits (g/day)	150	86 (32–136)	126 (76–175)
Fish (g/week)	70	0 (0–0)	0 (0–10)
Cereals, bread (g/day)	120	75 (50–103)	73 (53–97)
Pasta, rice, potatoes (g/day)	100	55 (36–84)	63 (43–89)
Milk, milk products (g/day)	330	280 (178–402)	351 (225–495)
Egg (g/week)	60	0 (0–12.5)	3.8 (0–18.3)
Meat, sausages (g/day)	35	37 (24–59)	34 (20–52)
Fat (g/day)	20	2.1 (0–4.9)	1.7 (0.4–5.3)
Sweets, snacks, soft drinks (1 portion/day) ^†^	1	2.3 (1.7–4.0)	1.6 (1.0–2.7)

Data are presented as the median (IQR). * According to the Optimized Mixed Diet for children in Germany [24]; ^†^ 10% of the total energy intake/day.

**Table 5:** Nutrient and food intake quality at nine and 24 months stratified by maternal educational level.

	Low Maternal Education *			High Maternal Education ^†^		
	BABYDIET Cohort	DONALD Cohort	*p*-Value ^‡^	BABYDIET Cohort	DONALD Cohort	*p*-Value ^‡^
9 months						
*n ^§^*	34	26		40	178	
Nutrient quality						
PANDiet	66.4 (57.9–73.2)	64.9 (59.9–68.0)	0.6	65.9 (56.3–71.0)	69.0 (63.2–73.4)	0.03
Added sugar (g/day)	8.8 (4.0–20.0)	7.9 (1.6–21.8)	0.5	3.6 (0.7–13.9)	1.4 (0.0–5.2)	0.01
Food quality						
SOCCER	73.3 (73.3–80.0)	93.3 (65.0–93.3)	0.1	73.3 (60.0–80.0)	93.3 (80.0–100.0)	<0.0001
24 months						
*n*	18	26		26	178	
Nutrient quality						
PANDiet	57.6 (52.8–62.5)	58.2 (55.1–62.8)	0.7	58.5 (55.8–61.1)	59.9 (55.4–63.6)	0.3
Added sugar (g/day)	28.8 (21.5–54.3)	21.0 (12.5–31.1)	0.05	22.7 (17.5–40.5)	17.5 (9.4–25.5)	0.006
Food quality						
HuSKY	47.6 (39.1–58.6)	56.0 (49.4–65.7)	0.07	53.7 (45.1–58.8)	58.6 (50.1–65.2)	0.01

Data are presented as median (IQR). * Low maternal education was defined as a secondary general or intermediate school certificate; ^†^ High maternal education was defined as a higher education entrance certificate; ^‡^ Mann-Whitney test; ^§^ Numbers are reduced because of missing data for maternal education. PANDiet: Probability of Adequate Nutrient Intake; SOCCER: Score Of Compliance to Complementary fEeding Recommendations; HuSKY: Healthy nutrition Score for Kids and Youth.

## Appendix A

**Table A1:** Calculation of the SOCCER score.

Meal Type	Includes Industrial Meals, Home-Made Meals, Food	Recommendation	Allocation of Points *
Vegetable-potato-meat dish (meals per day)	Industrial or home-made- Vegetable-potato-meat dish, Vegetable-potato-Fish dish, Vegetarian dish	1 per day	I/R < 1, points proportionally subtracted from 100; I/R ≥ 1, 100 points
Fruit-cereal dish (meals per day)	Industrial or home-made fruit-cereal dish OR fruits mixed with cereals OR bread with fruits	1 per day	I/R < 1, points proportionally subtracted from 100; I/R ≥ 1, 100 points
Milk-cereal dish	Industrial or home-made Milk-Cereal Dish OR Milk mixed with cereals OR bread with milk or cheese	1 per day	I/R < 1, points proportionally subtracted from 100; I/R ≥ 1, 100 points
Milk-dish	Breastmilk OR infant formula	1 per day	I/R < 1, points proportionally subtracted from 100; I/R ≥ 1, 100 points
Cow’s milk (dairy products)	Milk; cheese; yoghurt/curd/soured milk; cream cheese	Max. 200 mL/day	I/R ≤ 1, 100 points; I/R > 1, points proportionally subtracted from 100

I/R: intake of meal type (I)/recommended intake of meal type (R). SOCCER: Score Of Compliance to Complementary fEeding Recommendations; HuSKY: Healthy nutrition Score for Kids and Youth.
